# GADD153 as a novel molecular fingerprint for Sjögren’s syndrome

**DOI:** 10.1186/1878-5085-5-S1-A110

**Published:** 2014-02-11

**Authors:** Mahmood S Mozaffari, Jun Yao Liu, Rafik Abdelsayed, Babak Baban

**Affiliations:** 1 Department of Oral Biology, College of Dental Medicine, Georgia Regents University, Augusta, Georgia 30912, USA

## Scientific objective

Sjögren’s syndrome (SS) is a systemic autoimmune disease characterized by chronic focal leukocyte infiltration and inflammation of exocrine glands including salivary glands thereby resulting in persistent dry mouth and consequent increased risk for dental caries and periodontal disease. Assessment of leukocyte infiltration into minor salivary glands of lower lip biopsy samples is commonly used to aid with diagnosis of SS; however, this approach may identify 60-80% of those afflicted with the disease thereby requiring use of additional diagnostic tests/markers which relies on a better understanding of the pathogenesis of SS. Endoplasmic reticulum (ER) stress response is a pivotal regulator of inflammation and cell death. An integral component of ER stress-induced apoptosis is expression of the growth arrest- and DNA damage-inducible protein 153 (GADD153). Thus, we tested the hypothesis that SS is associated with increased salivary gland expression of GADD153 and increased leukocyte infiltration thereby contributing to inflammation and cell death.

## Technical approach/methods

We utilized the non-obese diabetic (NOD) mice, a model of SS-like disease, in association with immunostaining and flow cytometry-based studies.

## Results/interpretation

Salivary glands of NOD mice displayed a) increased GADD153 expression, b) foci of inflammatory cell infiltrates including CD3^+^ T and CD19^+^ B lymphocytes as well as M1 and M2 macrophages and c) increased pro-inflammatory interleukin (IL)-17 but reduced anti-inflammatory cytokine, IL-10. These changes were accompanied with disruption of mitochondrial membrane potential and significant increase in apoptosis and necrosis of salivary gland cells of NOD than control mice. To establish the relevance of GADD153 for the human condition, archived lower lip biopsy samples of non-SS subjects and those with a diagnosis of SS were subjected to immunostaining. The results show intense GADD153 immunostaining in biopsy samples of SS compared to non-SS subjects. Collectively, the results indicate that GADD153 regulates inflammation and cell death in salivary gland in SS.

## Outlook/expert recommendations

The present study has established the relevance and significance of GADD153 as a novel predictive and prognostic molecular fingerprint for SS. Further, assessment of minor salivary gland GADD153 expression could potentially be a more sensitive approach to help with diagnosis of SS, at early stage of the disease, than leukocyte infiltration. Importantly, subsequent studies should establish whether inhibition of GADD153 expression ameliorates signs and symptoms of SS for which effective treatment options remain elusive.

